# Bellevue Hospital Medical College

**Published:** 1861-07

**Authors:** 


BELLEVUE HOSPITAL MEDICAL COLLEGE.—Under this title a new school has recently been organized at New York. The faculty, as the accompanying card shows, consists of thirteen members, with a number of attaches, and the lectures are to be delivered at the hospital whose name the college bears.

JAMES R. WOOD, M D., Professor of Operative Surgery and Surgical Pathology.

FRANK H. HAMILTON, M.D., Professor of Military Surgery, Fractures, and Dislocations.

LEWIS A. SAYRE, M.D., Professor of Orthopedic Surgery.

ALEXANDER B. MOTT, M.D., Professor of Surgical Anatomy.

STEPHEN SMITH, M.D., Professor of the Principles of Surgery.

ISAAC E. TAYLOR, M.D., GEORGE T. ELLIOT, M.D., B. FORDYCE BARKER, M.D., Professors of Obstetrics and the Diseases of Women and Children.

BENJAMIN W. MCCREADY, M.D., Professor of Materia Medica and Therapeutics.

JOHN W. S. GOULEY, M.D., Professor of Descriptive and Microscopic Anatomy.

AUSTIN FLINT, M.D., Professor of the Principles and Practice of Medicine.

R. OGDEN DOREMUS, M.D., Professor of Chemistry and Toxicology.

AUSTIN FLINT, JR., M.D., Professor of Physiology

CHARLES D. PHELPS, M.D., Demonstrator of Anatomy.

N. R. MOSELY, M.D., Prosector to the Chair of Surgical Anatomy.

SYLVESTER TEATS, M.D., Prosector to the Chair of Operative Surgery and Surgical Pathology.

